# Paroxysmal Atrial Fibrillation in Elderly: Worldwide Preliminary Data of LINAC-Based Stereotactic Arrhythmia Radioablation Prospective Phase II Trial

**DOI:** 10.3389/fcvm.2022.832446

**Published:** 2022-03-02

**Authors:** Antonio Di Monaco, Fabiana Gregucci, Ilaria Bonaparte, Federica Troisi, Alessia Surgo, Domenico Di Molfetta, Nicola Vitulano, Federico Quadrini, Roberta Carbonara, Gaetano Martinelli, Pietro Guida, Maria Paola Ciliberti, Alba Fiorentino, Massimo Grimaldi

**Affiliations:** ^1^Department of Cardiology, General Regional Hospital F. Miulli, Bari, Italy; ^2^Department of Clinical and Experimental Medicine, University of Foggia, Foggia, Italy; ^3^Department of Radiation Oncology, General Regional Hospital F. Miulli, Bari, Italy; ^4^Department of Radiology, General Regional Hospital F. Miulli, Bari, Italy

**Keywords:** radioablation, stereotactic body radiotherapy, arrhythmia, atrial fibrillation (AF), elderly patients

## Abstract

Treatment approach for elderly patients with atrial fibrillation (AF) is difficult. The present prospective phase-II trial evaluated LINAC-based stereotactic arrhythmia radioablation safety in this population. The reported data of the first 5 patients worldwide, showed no side effects, absence of AF episodes and without antiarrhythmic drugs.

**Trial Registration:**

ClinicalTrials.gov, identifier: NCT04575662.

## Introduction

Atrial fibrillation (AF) is the most common cardiac arrhythmia affecting more than 40 million individuals in the world, and elderly age is a prominent risk factor (1).

AF increased the risk of stroke and heart failure with a reduction in functional capacity (1). Current guidelines recommend Pulmonary Veins (PVs) isolation with catheter ablation (CA) in symptomatic patients refractory to antiarrhythmic therapy (AAT) (1).

In elderly, paroxysmal AF is difficult to treat with drugs, since they alternate sinus bradycardia and fast rate AF in the so-called tachy-bradi syndrome, and by CA due to the higher complication rate (1, 2). Thus, a non-invasive approach should be favorite.

Other ablation approaches have been implemented in cardiac arrhythmia, including stereotactic arrhythmia radioablation (STAR) or radiosurgery which is a safe and effective arm in the oncological and non-oncological scenario. STAR, using high-dose radiation, produced great biological cell kill death by multifactorial results (DNA double-strand breaks, apoptosis, vascular damage, ischemic cell-death) (3–5).

As we reported in a previous review, in field of preclinical research applied to STAR, several studies were conducted in animal models, showed also in porcine model the PVs isolation to treat AF could be achieved by radiosurgery with a conventional LINear ACcelerators (LINACs) (3).

Several STAR data were published for ventricular tachycardia, using different technologies, including LINACs or Cyberknife, but, as reported recently by Lydiard et al., “LINACs have not yet been used for AF treatments” (6).

Based on the latter background, a prospective phase-II trial was designed to evaluate safety of LINAC-based STAR (ClinicalTrials.gov: NCT04575662). We selected the elderly population with refractory AF due to the risk of recurrence of FA after the standard CA procedure and due to the risk of complication for these patients.

Here, the data of the first 5 elderly patients worldwide were preliminary reported.

## Methods

Inclusion criteria were: age more than 70 years; symptomatic paroxysmal AF; intolerance or non-response to AAT. All patients performed 1-week ECG-Holter monitoring (ECG-HM) (during AAT and AAT wash-out), a complete transthoracic echocardiogram, including the left atrial (LA) strain evaluation before STAR.

The study was approved by the local Ethics Committee and all patients signed informed consent.

The Transthoracic echocardiogram and atrial strain evaluation were performed. For 2D-STE analysis, a volumetric image of LA from the apical view was obtained and stored. An echocardiography core laboratory measured parameters related to LA Strain with a dedicated software (Philips, Epiq 7-Auto Strain Tomtec Application). To assess LA Strain we evaluated LA Reservoir-Strain (LASr) based on ED (End-Diastole) and Pre-A (pre-Atrial contraction), LA Conduit-Strain (LAScd) based on ED and Pre-A, LA Contraction-Strain (LASct) based on ED and Pre-A. LA area strain analysis was performed according to the methods previously described and according to the Task Force to standardize deformation imaging (7).

**Figure 1:**
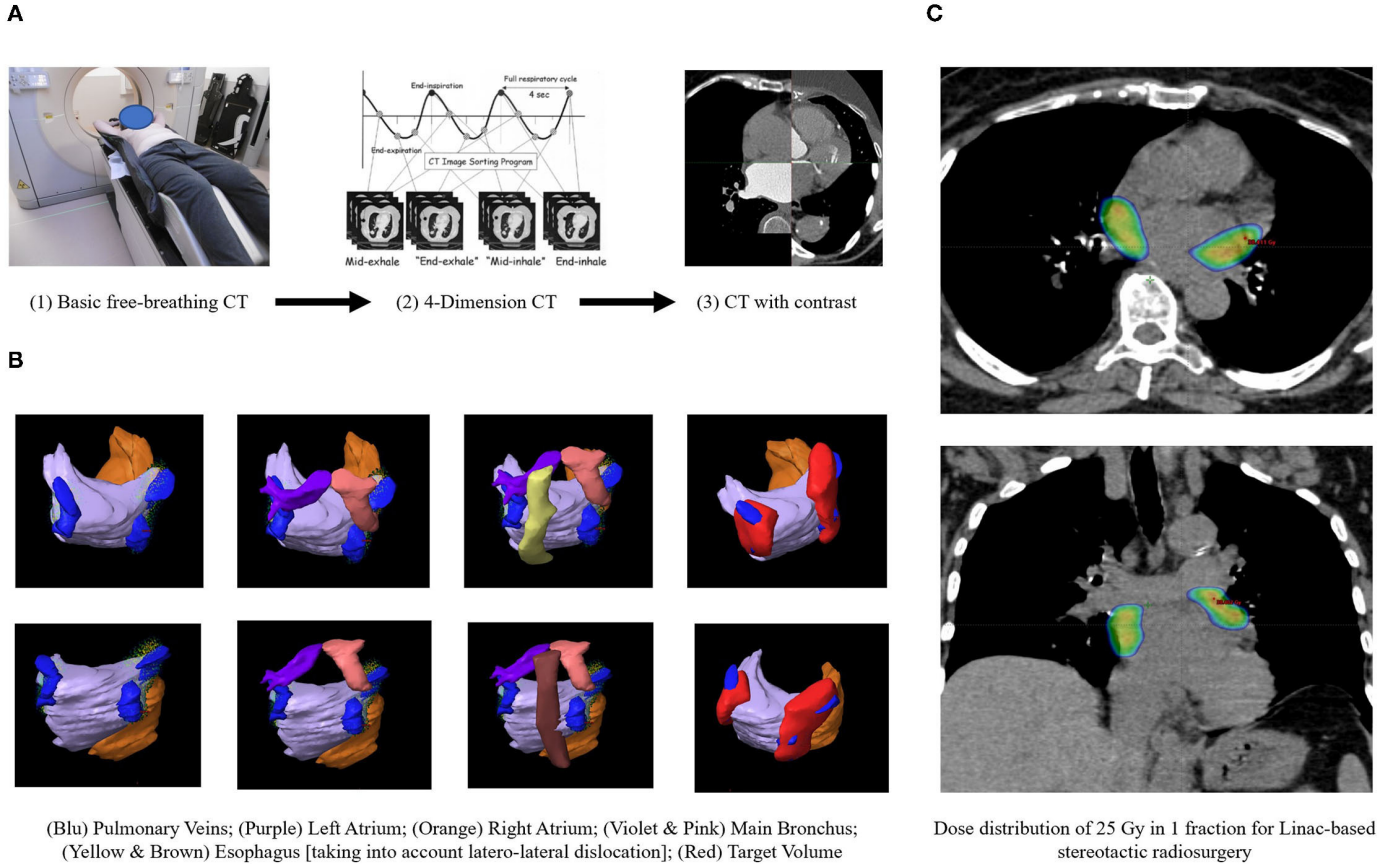
The workflow of LINAC-based STAR. **(A)** Simulation procedures. **(B)** Organs at risks and target volume identification. **(C)** Treatment planning.

STAR procedures were shown in Figure 1. They were immobilized using a vac-lock bag and 3 Computed Tomography (CT, 1 mm slice-thickness), in the supine position were performed: basic free-breathing CT for dose calculation; 4-Dimension CT for moving evaluation; CT with contrast for anatomical accuracy (3–5).

Several Organs at Risks (OaRs) were contoured, making more attention to esophagus and main bronchus, for which a planning risk volume (PRV) was built. The clinical target volume (CTV) was identified by radiation oncologists and cardiologist and was defined as the area around PVs. From CTV, an internal target volume (ITV) was created to compensate heart and respiratory movement. Finally, the planning target volume (PTV) was defined adding 0–3 mm to the ITV, excluding the overlap area with OaRs/PRV, where PTV was cropped.

STAR was performed in free-breathing with a PTV prescription total dose (Dp) of 25 Gy/1 fraction. A “simultaneous integrated protection” dose was realized to the interface between PTV-PRV to ensure the tolerability of critical structures (8). Flattening Filter Free (FFF), Volumetric Modulated Arc Therapy (VMAT) plan was generated, normalizing 100% Dp to 95% of the volume, while large intra-target dose heterogeneity D2% (PTV) <150%Dp was accepted. The treatment was generated, optimized and delivered by TrueBeam^TM^ (Varian Medical System). Image-guided radiotherapy (IGRT) with Cone Beam CT and Surface-Guided RadioTherapy (SGRT) with Align-RT (Vision RT) were used to reduce set-up error and to monitor patients during fraction.

Follow-up consisted of clinical evaluation during and for 48 h after STAR. One-week ECG-Holter monitoring, transthoracic echocardiogram and clinical evaluation are performed 1, 3, 6, and 12 months after STAR.

The primary endpoint is the 1-month post-STAR safety, as complete STAR delivery and no acute treatment-related adverse events more than G3, assessed according to the Common Terminology Criteria for Adverse Events (version 5.0). Secondary endpoints were: reductions in AF episodes and in AAT, overall survival. The sample size planning is 20 cases based on 95% success for the primary endpoint, with a significant level of 5% and a power of 90%.

## Results

From May 2021 to January 2022, 11 elderly patients were enrolled, of which 6 were treated. AAT was stopped after enrollment.

For primary endpoint (side effects at 1 month after STAR), 5 patients completed treatment without acute treatment-related adverse events (>G1) and 1 patient has only 7 days of follow-up. The main STAR data are summarized in Table 1. The treatment plan was delivered with 3 no-coplanar arcs, in all cases. The mean Overall Treatment Time (OTT) was 3 min. Only patient-1 needed adaptive real-live radiotherapy due to the esophagus position, in fact at IGRT before STAR the esophagus position was completely different from CT simulation.

**Table 1:** Patient characteristics and main treatment planning and dosimetric data.

	**PT1**	**PT2**	**PT3**	**PT4**	**PT5**
**Clinical Characteristics**					
Age (years)	77	70	82	89	79
Sex	Female	Female	Female	Male	Female
Cardiovascular risk factors	Dyslipidemia Hypertension	Dyslipidemia, Hypertension	Dyslipidemia, Hypertension	Hypertension	Hypertension
Other pathologies		Anxiety disorder Dysthyroidism	Dysthyroidism	Chronic Bronchitis, mild renal insufficiency	Dysthyroidism
Body Mass Index (Kg/m^2^)	27	24	26	27	27
Time of onset of AF (years)	10	2	15	3	30
Maximum AF duration (hours)	10	14	12	24	24
Symptoms	Palpitations Lipothymia	Palpitations, Dyspnea	Palpitations	Palpitations, lipothymia	Palpitations, Dyspnea
1 week ECG Holter monitoring before radiotherapy (AAT wash-out)	7 AF episodes (max duration 11 min, mean ventricular rate 128b pm)	4 AF episodes (max duration 60 min, mean ventricular rate 138 bpm)	2 AF episodes (max duration 7 h, mean ventricular rate 142 bpm)	11 AF episodes (max duration 15 h, mean ventricular rate 171 bpm)	4 AF episodes (max duration 9 h, mean ventricular rate 153 bpm)
EHRA symptom scale	2b	2b	2b	2b	2b
Drug Therapy at enrolment	Atenolol 25 mg^*^ Flecainide 200 mg^*^ Apixaban 30 mg	Rivaroxaban 20 mg Amiodarone 200 mg^*^ Bisoprololo 1.25 mg^*^ L-thyroxine 50 mcg Olanzapine 2.5 mg Ramipril 2.5 mg Furosemide 25 mg Synvastatin 20 mg	Dabigatran 110 mg Losartan 50 mg Synvastatin 20 mg Ezetimibe 10 mg L-thyroxine 50 mcg	Bisoprolol 2.5 mg^*^ Amiodarone 200 mg^*^ Furosemide 25 mg Warfarin 5 mg	Flecainide 200 mg^*^, L-thyroxine 75 mcg Bisoprolol 2.5 mg^*^, Olmesartan 40 mg, rivaroxaban 20 mg, doxazosin 2 mg
**Radiotherapy parameters**					
CTV	15.4 cc	11.3 cc	25.3 cc	15.86 cc	15.8 cc
ITV	36 cc	33.2 cc	37.8 cc	44.5 cc	32.6 cc
PTV	53.5 cc	52.1 cc	59.1 cc	56.6 cc	49 cc
Prescription isodose	79%	75%	73%	74%	73%
D2%	30 Gy	31.8 Gy	31.9 Gy	32.6 Gy	32.2 Gy
Maximum dose to esophagus	13.8 Gy	13.2 Gy	11.8 Gy	13.2 Gy	15.4 Gy
Maximum dose to left bronchus	15.8 Gy	12.2 Gy	18.2 Gy	19 Gy	10.2 Gy
Maximum dose to right bronchus	13.7 Gy	20.6 Gy	4.5 Gy	5.9 Gy	8.5 Gy
Mean dose to heart minus PTV	5.2 Gy	3.7 Gy	4.8 Gy	4 Gy	3.7 Gy
OTT	3 min	3 min	3 min	3 min	3 min

* *Drug stopped after enrollment. AAT, antiarrhythmic therapy; AF, atrial fibrillation; CTV, clinical target volume; EHRA, European Heart Rhythm Association; ITV, internal target volume; PRV, planning risk volume; PTV, planning target volume; OTT, Overall Treatment Time*.

Four patients completed a 3 months FUP with transthoracic echocardiogram data. At baseline, LAV and LAA in PT-1 were significantly increased, in PT-2 moderately and in PT-3 slightly compared to the reference values. Strain parameters at baseline were also mild to moderate compromised or severely compromised compared to the reference values (Table 2).

**Table 2:** Main echocardiographic and ECG-HOLTER parameters.

	**PT1**			**PT2**			**PT3**			**PT4**		
	**BAS**	**1M**	**3M**	**BAS**	**1M**	**3M**	**BAS**	**1M**	**3M**	**BAS**	**1M**	**3M**
LAV (ml)	64.2	62.7	61.5	97.8	83	81.4	68.1	63.5	50.3	94	92.3	90.4
LAA (cm^2^)	20.9	21.5	22	28.5	25.6	25.1	20.1	21	18.2	26	25.8	24.9
LASr (ED)	13.2%	9.1%	15.4%	16.7%	13.3%	16%	27.7%	27.7%	22%	20%	16.5%	21.2%
LAScd (ED)	−8.2%	−5.6%	−4.5%	−9%	−9.7%	−4.5%	−8.2%	−6.7%	−5.8%	−16.2%	−15.4%	−12.9%
LASct (ED)	−5%	−3.5%	−11%	−7.7%	−3.6%	−11.5%	−19.5%	−21.1%	−16.2%	−7.9%	−1.1%	−8,2%
LASr (preA)	12.5%	8.8%	13.9%	15.5%	12.9%	14.3%	23%	22.9%	18.9%	21%	16.3%	20%
LAScd (preA)	−7.8%	−5.5%	−4%	−8.3%	−9.4%	−4%	−6.9%	−5.5%	−5%	−15.7%	−15.3%	−12.2%
LASct (preA)	−4.8%	−3.4%	−9%	−7.2%	−3.5%	−10.3%	−16.3%	−17.4%	−13.9%	−7.3%	−1.1%	−7.8%
LVEF (%)	55	55	58	55	55	55	55	55	58	45	45	49
Mitral valve regurgitation	Mild-Moderate	Mild-Moderate	Mild	Moderate	Moderate	Mild-moderate	Mild-Moderate	Mild-Moderate	Mild-moderate	Mild-moderate	Mild-moderate	Mild-moderate
Aortic valve regurgitation	-	-	-	Mild	Mild	Mild	Moderate	Moderate	Moderate	mild	mild	mild
Tricuspid valve regurgitation	Mild	Mild	Mild	Moderate severe	Moderate	Mild-moderate	Mild-Moderate	Mild-Moderate	Mild	Mild-moderate	Mild-moderate	Mild-moderate
Pericardial effusion	-	-	-	-	-	-	-	-	-	-	-	-
1-week ECG-Holter monitoring performed 1-month after STAR Data												
	PT1			PT2			PT3			PT4		
atrial ectopy	89,642 beats			27,675 beats			20,305 beats			58,004 beats		
Atrial	19 episodes, max duration			73 episodes, max duration			5 episodes, max duration			26 episodes, max duration		
tachycardia	2 h, meanVR 160 bpm			90 min; meanVR 146 bpm			12 min; meanVR 158 bpm			140 min; meanVR 147 bpm		

*ED, end diastole; LAA, center atrial area; LAScd, center atrial conduit strain; LASct, center atrial contraction strain; LASr, center atrial Reservoir strain; LAV, center atrial volume; LVEF, center ventricle ejection fraction; Pre-A, pre-atrial contraction; VR, ventricular rate*.

The 1-week ECG-HM performed 1-month after procedure documented frequent atrial ectopy and atrial tachycardia without AF recurrences. A rare atrial ectopy without AF recurrences was documented at 3 months after procedure. No patients started AAT after radiotherapy. Regarding echocardiographic data, at 1-month follow-up in PT1-2-4 LASr (ED) and LASr (pre-A) were reduced with a prevalent reduction of the LASct component. In the PT-3, no significant reduction in the strain parameters was reported. At 3-month, a recovery trend of the global strain parameter was shown in PT1-2-4. At 1 and 3-months LAV and LAA were slightly reduced in all patients.

Patients 1–3 had a FUP of 6–7 months, Patient 4 a FUP of 3 months, Patient 5 a FUP of 1 month. For all treated patients with a mean follow-up of 4 months, no acute and late side effects were reported. Only one patient experienced G1 esophagitis (7 days from STAR), improved by 5 days of medical therapy.

## Discussion

Elderly patients affected by AF are a fragile population at higher risk of all CA procedural complications (1, 2), including vascular injury, cardiac perforation, phrenic nerve injury, stroke, and most concerning, atrio-esophageal fistula, which portend a high mortality rate and a higher rate of AF recurrences. For the latter reasons, in the clinical practice it is preferred to use pharmacological treatment rather than interventional procedures to treat AF in elderly.

STAR approach have been recently implemented, but no experience of LINAC has been published for AF (6). The present phase-II preliminary worldwide LINAC-STAR data on elderly patients showed: no acute toxicities; no AF episodes; no AAT use.

Three STAR-AF cases were published with Cyberknife technology, reporting an OTT of 90 min (9, 10). To optimize target tracking during cardiorespiratory motion, an internal fiducial marker was placed transvenously in proximity to the left atrial target (9). In 2 out 3 patients, AF occurred at 6-months from Cyberknife-STAR (9, 10).

Comparing the latter data with the present analysis, 2 differences should be highlighted.

In regards to target volume, the mainstay AF ablation approach is a PVs isolation, while appropriate/effective ablation targets, including atrial wall, remain poorly defined (1, 9, 10). In the Cyberknife cases, PVs and the left atrial posterior wall were irradiated, while in the present study, target was defined as the area around PVs. Moreover, higher dose was mainly located on the left lateral ridge, the area between appendage and left PVs (higher arrhythmogenesis area) (11).

In terms of TT, Cyberknife device is mounted on a robotic arm to deliver radiation to a tumor from different trajectories, while LINAC with a rotation of its gantry deliver high dose of radiation in a shorter time (3 vs. 90 min) (12). However, the shorter time is essential for reducing intrafraction motion, so in the present trial, due to the motion study and IGRT/SGRT monitoring, the introduction of fiducial was not necessary (3–5, 12).

Cyberknife device is mounted on a robotic arm to deliver radiation to a tumor from different trajectories, while LINAC with a rotation of its gantry deliver high dose of radiation in a shorter time (3 vs. 90 min).

Due to the innovation of this treatment for AF, sufficient data regarding procedural complications are not still available. However, previous studies reported STAR related complications during ventricular tachycardia treatment. In particular, a low percentage of complications was reported mainly within 90 days from STAR (heart failure exacerbation, radiation pericarditis and pneumonitis, nausea) and at long term follow up (mitral valve regurgitation worsening, pericardial effusions and gastro-pericardial fistula) (12).

Moreover, this analysis is the first to report clinical response. The frequent atrial ectopy and the atrial strain variations after procedure are probably indirect signs of effective atrial irritation due to radiotherapy. Some recent studies have shown the correlation between LA strain and atrial fibrosis, hypothesizing its prediction of the usefulness of ablation procedure (13). In this study, LAS parameters, based on ED and Pre-A, in three patients reduced 1-month after STAR. The LASct decreasing is related to atrial contraction phase of the cardiac cycle, probably due to acute irritation of the atrium immediately after STAR. The patient-3 showed no significant reduction in LAS parameters in the 1-month follow-up probably because atrial strain values were slightly reduced compared to normal. At the 3-month, a trend in recovery of LASr was found in the three patients. Finally, a reduction trend in LA volume and area was found in all patients probably because the maintenance of sinus rhythm improved ventricular performance with a reduction in the extent of mitral and tricuspid insufficiency and LA volume overload.

## Conclusion

The present collected data are promising, showing the safety of LINAC-based STAR for AF for the first 5 patients. This new ablation approach could represent a valid non-invasive alternative for elderly who were excluded from catheter ablation. Prospective randomized trials are guaranteed.

## Data Availability Statement

The raw data supporting the conclusions of this article will be made available by the authors, without undue reservation.

## Ethics Statement

The studies involving human participants were reviewed and approved by Comitato Etico Interregionale; Policlinico di Bari - Bari. The patients/participants provided their written informed consent to participate in this study.

## Author Contributions

AD, FG, AF, and MG write the protocol study and have the idea. IB, FG, AS, RC, MC, and AF are the responsibility of radiotherapy and radiotherapy data. AD, MG, NV, FQ, and FT are responsible of cardiological data. GM and DD are responsible of radiological data. AD, FG, and AF write the paper. PG analyzed the statistical data. All authors corrected, improved the manuscript, and interpreted the results.

## Conflict of Interest

The authors declare that the research was conducted in the absence of any commercial or financial relationships that could be construed as a potential conflict of interest.

## Publisher's Note

All claims expressed in this article are solely those of the authors and do not necessarily represent those of their affiliated organizations, or those of the publisher, the editors and the reviewers. Any product that may be evaluated in this article, or claim that may be made by its manufacturer, is not guaranteed or endorsed by the publisher.
